# An open invisible space enabled by reconfigurable metasurfaces and self-play reinforcement learning

**DOI:** 10.1038/s41377-025-01944-5

**Published:** 2025-09-15

**Authors:** Xinman Yin, Yanyu Zhao

**Affiliations:** Beijing Advanced Innovation Center for Biomedical Engineering, Key Laboratory for Biomechanics and Mechanobiology of Ministry of Education, School of Engineering Medicine, Beihang University, Beijing, 100191 China

**Keywords:** Optoelectronic devices and components, Displays

## Abstract

An open, dynamic, and electromagnetically invisible space has been constructed using reconfigurable metasurfaces and self-play reinforcement learning. A model named MetaSeeker is proposed to optimize the cloaking performance of randomly distributed metasurfaces. The hidden objects can move freely within the constructed invisible space, with environmental similarity of 99.5%. This advancement provides an innovative solution for cloaking technologies in complex environments.

**Figure:**
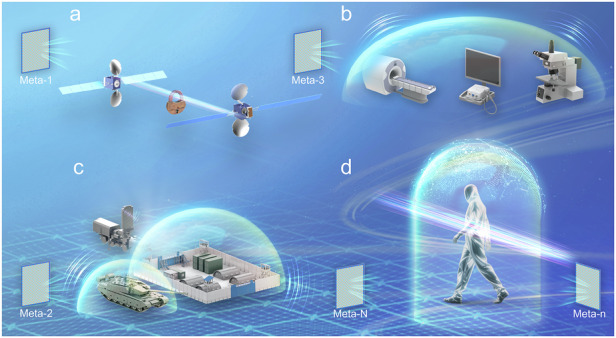
An open invisible space enabled by reconfigurable metasurfaces and self-play reinforcement learning.

An open invisible space enabled by reconfigurable metasurfaces and self-play reinforcement learning.

Flexible control of electromagnetic (EM) waves to make objects invisible has promising applications across various fields, including protecting military equipment, stabilizing wireless communication, and encrypting sensitive signals. In recent years, with the advent of metamaterials and metasurfaces^1–4^, many cloaking methods have been successively proposed, including transformation optics^5^, near-zero refractive index^6^, scattering cancellation^7^, and reconfigurable metasurface technologines^8,9^. However, these methods typically construct a static, closed space that fails to satisfy open-environment requirements, and are restricted to predefined objects and idealized environments^10,11^, resulting in limited generalization capabilities. These limitations highlight the necessity of rendering an open and cluttered space invisible.

To meet the demands of practical applications, in a newly published paper^12^ in *Light: Science & Applications*, Bei Wu, Chao Qian, Zhedong Wang, Pujing Lin, Erping Li, and Hongsheng Chen from Zhejiang University have reported an open, dynamic, EM invisible space in complex environments. As shown in Fig. 1, this method renders an invisible space by deploying a team of randomly distributed metasurfaces. A self-play artificial intelligence system named MetaSeeker is developed for optimized control of the metasurfaces. Traditional inverse design of metasurfaces typically relies on deep learning, which requires substantial human efforts to construct a training dataset and often fails to generalize beyond the scope of the constructed dataset. This limitation becomes particularly prominent when addressing high-degree-of-freedom optimization problems. To address the ultra-high-dimensional optimization challenges (with complexity reaching 10^96^) inherent to open dynamic systems, the research team proposed MetaSeeker, a population-based reinforcement learning (RL) model. MetaSeeker utilizes a population of RL agents to autonomously interact with the environment for sample collection and conduct on-site learning based on far-field feedback, ultimately achieving continuous cloaking performance enhancement without human intervention. This work breaks the limits of conventional cloaking methods and paves the way for EM cloaking technology in real-world scenarios.

**Fig. 1:**
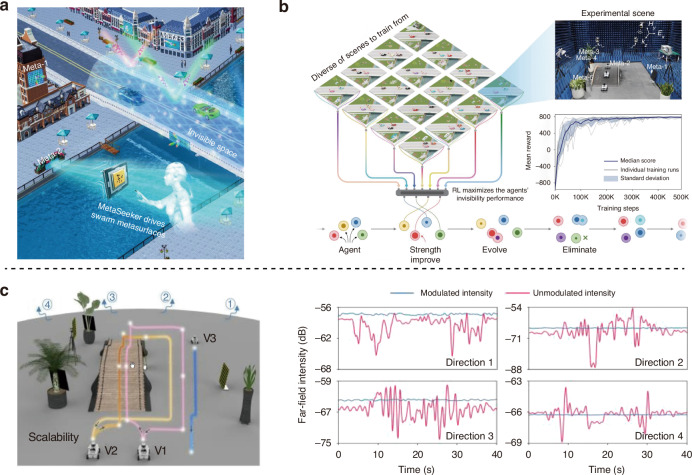
MetaSeeker: sketching an open invisible space. **a** An EM invisible space is created through a team of metasurfaces. A swarm of reconfigurable metasurfaces is mastered by the MetaSeeker. **b** Two-tier optimization strategy adopted for incorporating model-based RL and population-based training. **c** Demonstration of EM invisibility under dynamic scenarios. The subfigures are cropped from the corresponding reference

MetaSeeker adapts a two-tier optimization approach by incorporating model-based RL and population-based training (PBT)^13^. The PBT framework dynamically adjusts the hyperparameters of the RL process by eliminating underperforming agents. This autonomous process aligns seamlessly with Darwin’s theory of evolution and eliminates the need for human intervention. Specifically, MetaSeeker integrates four interconnected components for recognition, representation, dynamics, and prediction. The model maps the real physical world to the virtual internal world of agents, where the agents can autonomously optimize, evolve, and adapt. Through this process, each agent’s cloaking performance significantly enhances until it reaches a state of saturation.

To verify the scalability of the invisible space, the researchers conducted experiments in a microwave anechoic chamber, operating at 5 GHz. The experiment demonstrated the dynamic process of adding a third vehicle to a moving formation of two vehicles. The results showed the variation of far-field intensity over time during the vehicles’ movement, with cloaking performance reaching 99.73%, 99.74%, 99.77%, and 99.95% across four detection angles, respectively. The experimental results validated the system’s strong resistance to interference, demonstrating stable cloaking effectiveness across different disturbance conditions.

Creating an open, dynamic, and EM invisible space holds transformative potential across various fields, including encryption of satellite communications, shielding of external EM interference to improve the signal-to-noise ratio of imaging systems^14^, protection of military facilities^15^, and extension from microwave frequencies to higher frequencies (such as visible, near-infrared, and shortwave infrared bands), as illustrated in Fig. 2^16,17^. It is noted that these potential applications face certain challenges. For example, as the required number of metasurfaces increases, the complexity of optimization would grow exponentially, making conventional algorithms inadequate for real-time demands. In addition, although the design principles can be generalized to higher frequencies following a similar manner, a key challenge in optical invisibility experiments, as compared to microwave invisibility, arises from the intricate fabrication processes required for large-scale optical metasurfaces, coupled with their heightened sensitivity to error fluctuations^18–20^.

**Fig. 2:**
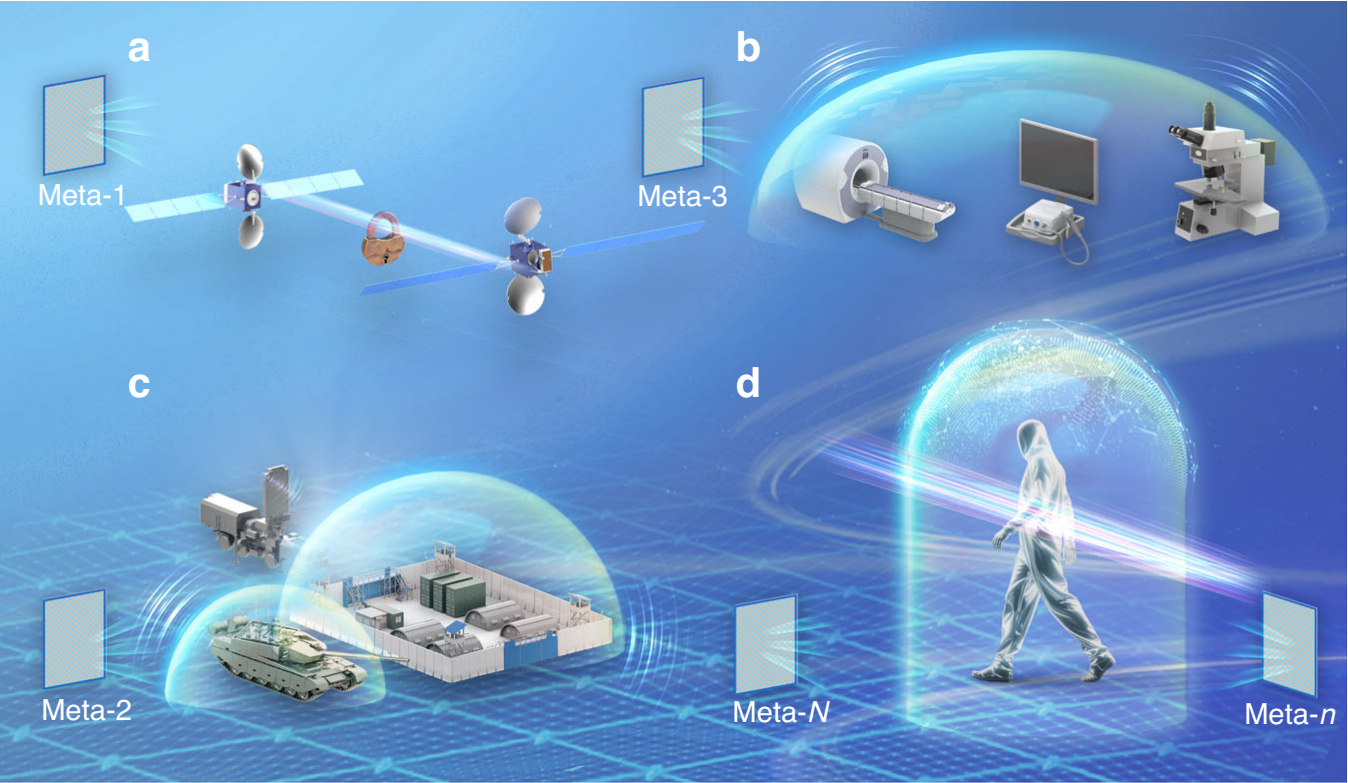
Application prospects for an open, dynamic, and EM invisible space. **a** Encryption of satellite communications. **b** Shielding of external EM interference to improve the signal-to-noise ratio of imaging systems. **c** Protection of military facilities. **d** Cloaking in higher frequencies such as visible, near-infrared, and shortwave infrared bands

Looking forward, with future advancements in intelligent algorithms and precision manufacturing, the EM cloaking technology will ultimately develop toward full-spectrum invisibility and enable real-world applications across various fields such as communication, defense, and mass consumption.
